# Sustainable Applications of Thymol: Advances in Formulation Technologies, Drug Delivery Systems, and Food Preservation Strategies

**DOI:** 10.1002/fsn3.71779

**Published:** 2026-04-25

**Authors:** Farhang Hameed Awlqadr, Mohammed N. Saeed, Ammar B. Altemimi, Syamand Ahmed Qadir, Aryan Mahmood Faraj, Othman Abdulrahman Mohammed, Tablo H. Salih, Rawaa H. Tlay, Tarek Gamal Abedelmaksoud

**Affiliations:** ^1^ Food Science and Quality Control, Halabja Technical College, Sulaimani Polytechnic University Sulaymaniyah Iraq; ^2^ Department of Nutritional Analysis and Health, Kifri Technical College, Garmian Polytechnic University Kifri Sulaimaniyah Iraq; ^3^ Department of Food Science, College of Agriculture University of Basrah Basrah Iraq; ^4^ Medical Laboratory Techniques Department, Halabja Technical Institute, Research Center, Sulaimani Polytechnic University Sulaymaniyah Iraq; ^5^ Medical Laboratory Science Department, Halabja Technical College Sulaimani Polytechnic University Sulaymaniyah Iraq; ^6^ Department of Food Science, Faculty of Agricultural Engineering Damascus University Damascus Damaskus Syrian Arab Republic; ^7^ Food Science Department, Faculty of Agriculture Cairo University Giza Egypt

**Keywords:** antimicrobial activity, bioactive compound, drug delivery, edible coatings, intelligent packaging, nanotechnology

## Abstract

Thymol, a monoterpene phenol derived from *Thymus vulgaris* , suppresses a wide range of microorganisms and possesses antioxidant, anti‐inflammatory, and anticancer effects. This article focuses on the biomedical and dietary applications of thymol and briefly covers its physiological mechanisms and administration routes. Aqueous solubility limitations, high volatility, and oxidative instability hinder the practical applications of thymol. Nanoemulsions, liposomes, micelles, and polymeric nanoparticles have been widely used to stabilize, solubilize, and make thymol more accessible to the body. Drug delivery to the target location can be controlled to some extent and may improve targeting efficiency, maximizing therapeutic effectiveness and minimizing unwanted effects. Thymol is a safe, effective, and environmentally sustainable food preservative, a film‐forming agent, and a key ingredient in new packaging solutions. This is because its antimicrobial activity prevents the growth of pathogenic bacteria, extends shelf life, and preserves the sensory quality of meat, dairy, fruits, and vegetables. Despite strong trends, progress, and research, large‐scale clinical studies are still lacking, and the regulatory paths are unclear. To meet this challenge, clinical validation should lead to scientific, pharmacological, transdisciplinary, and sustainable production techniques. Thymol is a versatile and sustainable natural substance that can be used to improve food safety and therapeutics. Overcoming existing difficulties will allow this product to be used commercially and ensure sustainable natural health and food preservation solutions.

## Graphical abstract

Thymol is a natural monoterpene with antimicrobial, antioxidant, anti‐inflammatory, and anticancer activities relevant to food and biomedical applications. Its poor solubility and instability can be improved through nanodelivery systems. Thymol also supports sustainable food preservation and active packaging, although further clinical validation and clearer regulatory frameworks are needed for wider commercialization.

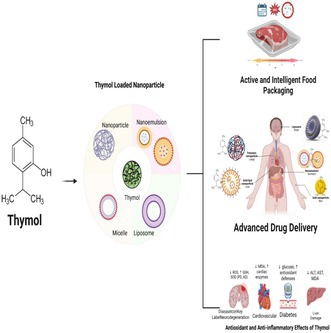

## 1 Introduction

Thymol, chemically known as 2‐isopropyl‐5‐methylphenol, is a natural monoterpene phenol derivative that is the main component responsible for its typical aromatic odor and antimicrobial activity. The main historical source of thymol, particularly from the genus Thymus species and *Thymus vulgaris* , has been in the last century in Europe. Because of its effectiveness as an antimicrobial, antioxidant, anti‐inflammatory, and preservative, thymol has been used in the past for the treatment of respiratory symptoms and digestive disorders, as well as a preservative agent to prolong the shelf life of food (Bouftou et al. 2025; Salehi et al. 2018). From a chemical perspective, thymol has a phenolic structure with hydrophobicity, crystallinity, and limited aqueous solubility. Such properties not only define thymol as a potent active compound, but also its action mechanisms by which it interacts with biological membranes as well as with microorganisms. Hydrophobicity enables thymol to be lodged deep into lipid bilayers, which eventually causes the rupture of cell integrity of the microorganisms and hence strong antimicrobial activity (Gago et al. 2025; Phyo et al. 2025). In addition, the phenolic hydroxyl of thymol is most likely the center of its antioxidant activity through which it very well can scavenge the radicals, reactive oxygen species (ROS), and, thereby, inhibit the cascade of oxidative damage to the cells in the body.

In scientific studies, the mechanistic aspects of the chemical properties of thymol are cited as the starting point of laborious industrial applications in new pharmaceutical formulations, innovative drug delivery routes, and sustainable food preservation technologies. According to the latest scientific developments, research has focused on different encapsulation protocols to achieve easy pharmaceutical and food integration, as well as delivery to targeted sites for better release profiles and therapeutic applications. For instance, the various microencapsulation and nanoemulsion strategies can significantly enhance the stability, solubilization, and release profiles of thymol while overcoming its drawbacks at such volatile and hydrophobic nature (Đorđević et al. 2025; Ozogul et al. 2025).

Nanotechnology has greatly broadened thymol as a therapeutic agent, with potential for site‐specific delivery and long retentions. Polymorphous investigations of thymol have proved that the drug can be effectively encapsulated in lipid‐based carriers, polymeric nanoparticles and nanoemulsions; as a result, the therapeutic potential of thymol for various diseases including bacterial infections, inflammation, and cancer is significantly increased. Besides fixing the drug availability issue, such systems make the biological part of the drug more effective and meanwhile lessen side effects which are often experienced with usage of synthetic pharmaceuticals (Julekha et al. 2025; Kashi et al. 2025). In addition, thymol is often used as a natural preservative and antimicrobial in the food industry. This is due to the fact that a substance is extremely active in terms of both antibacterial and antifungal effects Thymol exhibits proven antimicrobial activity against pathogenic bacteria such as *Escherichia coli* and *Staphylococcus aureus* , as well as fungi responsible for food spoilage. Recent advances in active packaging systems incorporating thymol‐loaded films have demonstrated effectiveness in extending shelf life, enhancing food safety, and reducing the reliance on synthetic preservatives in a cost‐effective and sustainable manner (Bouftou et al. 2025; Diao et al. 2025; Zhu et al. 2019).

The transformation of traditional applications into a number of new technological ones serves to illustrate the adaptability of thymol and its significance for scientific investigations. The use of pH‐responsive films and nanoencapsulated formulations in smart packaging materials is a clear representation of its potential to revolutionize food preservation methods by preserving the quality and safety of food while being environmentally friendly (Falleh 2025; Rojas et al. 2023). Owing to the wide and varied use of thymol, the current review aims to analyze in detail the recent scientific development of pharmaceutical formulations, drug delivery technologies, and food preservation that use the bioactivities of thymol. This review aims to reveal the therapeutic potential of thymol, identify gaps in the research area, and delineate future tendencies for industrial and biomedical applications by means of a thorough and critical evaluation of the available research literature. Primarily, this research intends to equip the scientific community, industry players, and policymakers with enlightening data that can facilitate the progress of innovations based on thymol and thus be consistent with the global call for sustainable healthcare solutions and food safety practice enhancements.

## 2 Bioactivity and Pharmacological Properties of Thymol

### 2.1 Antimicrobial Activities

Thymol is a phenolic monoterpene that comes mainly from *Thymus vulgaris* and *Origanum vulgare* and has been drawing a lot of scientific attention recently owing to its wide‐ranging antimicrobial effects as well as its possible therapeutic value. Its key mechanism of action, which prominently features membrane disruption of microbial cells leading to increased permeability, at the same time loss of membrane potential, and cell death, has been described in several reports. Being amphipathic, this molecule enters the lipid bilayers due to its hydrophobic nature, resulting in a disruption of the membrane structure and the occurrence of membrane proteins and ion gradients that are slightly damaged (Salehi et al. 2018; Waheed et al. 2024). For example it was shown that thymol disrupted the NADPH/NADP^+^ balance in *Staphylococcus aureus* , and there was lipid peroxidation as well as membrane rupture, Li et al. (2022), whereas Wang et al. (2022) revealed by electron microscopy that membranes had holes in *Zygosaccharomyces rouxii* after treatment with thymol. The physical changes described here entail mitochondrial dysfunction, excessive generation of reactive oxygen species, and disturbance of DNA and protein synthesis, thus making thymol a multimodal antimicrobial (Ahmad et al. 2021; Aljaafari et al. 2021).

**FIGURE 1:**
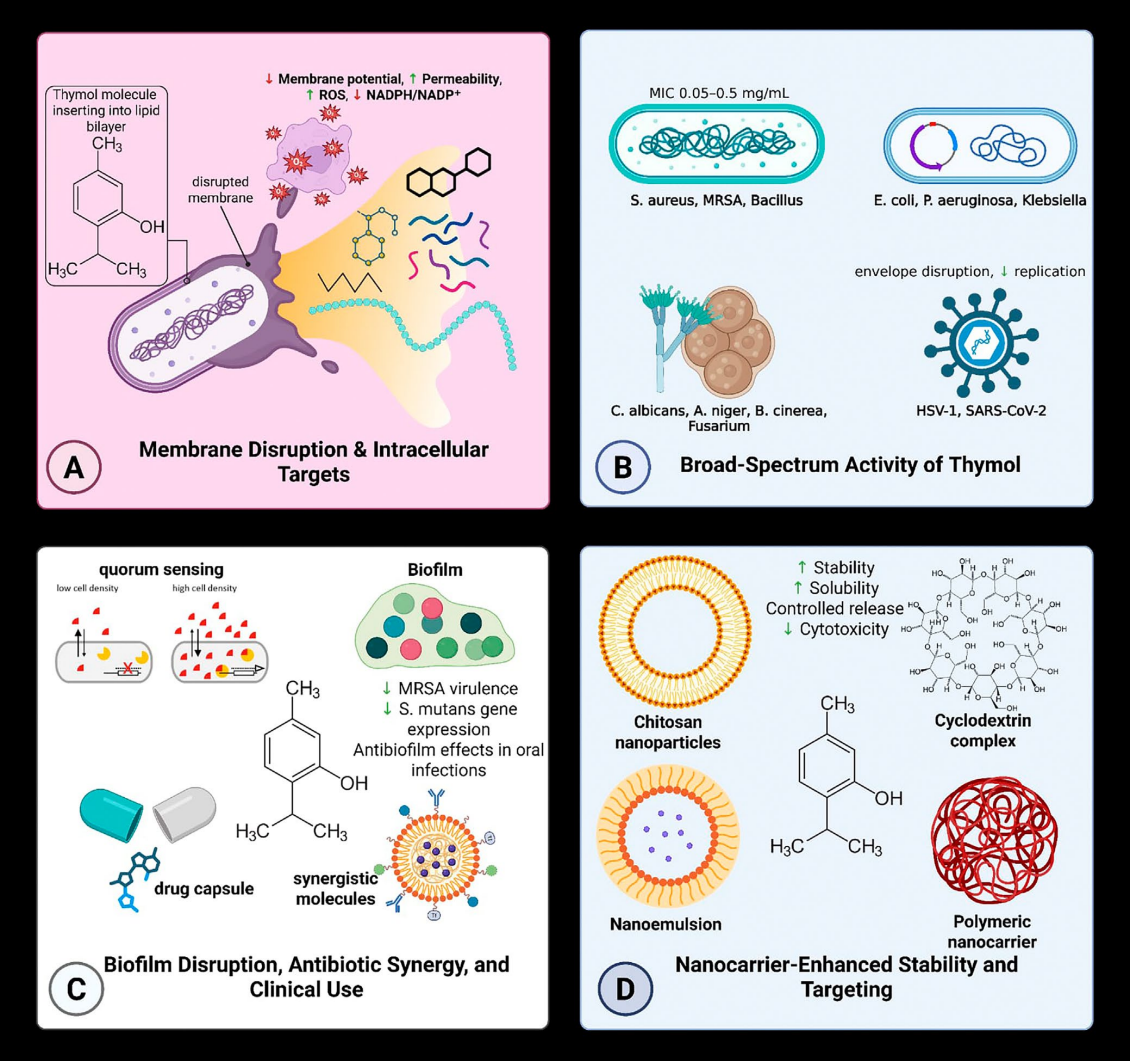
Mechanisms underlying the antimicrobial activity of thymol. Thymol disrupts microbial cell membranes and intracellular processes, increasing permeability and reactive oxygen species (ROS) production (A). It exhibits broad‐spectrum activity against bacteria, fungi, viruses, and yeasts (B), inhibits biofilm formation and quorum sensing, and shows synergistic effects with antibiotics (C). Its stability and bioavailability can be enhanced using nanocarrier systems such as nanoparticles, nanoemulsions, cyclodextrins, and polymeric carriers (D).

The antimicrobial activity of thymol extends to a wide variety of pathogens, and is effective against bacteria, fungi, viruses, and yeasts. This compound effective against both Gram‐positive and Gram‐negative bacteria, as it can penetrate their cells, but Gram‐positive bacteria that consist of less complex membranes, such as *Staphylococcus aureus* and *Bacillus cereus* , are more sensitive to thymol (Memar et al. 2017; Trombetta et al. 2005). For *S. aureus* , the MIC values for thymol investigated was ranged from 0.05 to 0.5 mg/mL and from 0.2 to 0.4 mg/mL (Li et al. 2022) and *E. coli* 0.2–0.4 mg/mL (Marchese et al. 2018), which did not cause structural damage to cell membrane as indicated with *E. coli* cellular linings, reported by Valliammai et. (VP20). Increased antimicrobial potential against methicillin‐resistant *S. aureus* (MRSA) (Valliammai et al. 2020). Moreover *Listeria monocytogenes* (Friedman et al. 2002), *Salmonella typhimurium* (Burt 2004), *Pseudomonas aeruginosa* (Gavanji et al. 2015), *Klebsiella pneumoniae* (Rani et al. 2022), and *Streptococcus mutans* (Kashi et al. 2025). Collectively, these data demonstrate the efficacy of thymol against a variety of pathogenic bacteria. The mechanism of action for this compound are shown in Figure 1 (membrane disruption, oxidative stress induction, and quorum sensing inhibition).

Thymol also exhibits significant antifungal activity through mechanisms similar to those observed in bacteria. It has been reported to inhibit *Candida albicans* by reducing ergosterol content and inducing reactive oxygen species (ROS) accumulation (Khan et al. 2015). Similarly, thymol disrupts membrane integrity and lipid composition in *Botrytis cinerea*, further confirming its antifungal efficacy (Zhang, Ma, et al. 2019). The effect of thymol led to abnormal hyphal morphology and cytoplasmic leakage in *Fusarium oxysporum* (Hao et al. 2023). Furthermore, strains such as *Aspergillus niger* (Bouddine et al. 2012), *Penicillium expansum* (Wang et al. 2016), and *Cryptococcus neoformans* (Stan et al. 2021). Yeasts are highly sensitive to thymol, such as *Zygosaccharomyces rouxii*, where MIC values can be as low as 0.0625 mg/mL (Wang et al. 2022), indicating its fungistatic and fungicidal properties. The SEM data support the presence of damage to the membrane as the cause of morphological changes (Zhang, Ma, et al. 2019). Moreover, thymol may be a potential antiviral agent, which is a new and promising field of study. Thymol effectively prevents herpes simplex virus type 1 (HSV‐1) in Vero cells by disrupting viral envelope function (Astani et al. 2010). Another study recently explored nanoformulated thymol against SARS‐CoV‐2, stating that it disrupted the viral entry and replication processes by breaking down lipids in the virus (Seadawy et al. 2020).

At the clinical level, thymol has demonstrated potential as either a supplementary or substitute agent against synthetic antimicrobials. The majority of scientific investigations point to thymol bringing about similar or even greater results than those of typical antibiotics such as tetracycline, gentamicin, and chloramphenicol. One such example is the research of Jeong et al. (2024) showed that thymol disrupted polymicrobial biofilms of *S. aureus* and *Candida albicans* , which are usually resistant to conventional treatments. Hyldgaard et al. (2012) corroborated these findings by stating that thymol allows antibiotics to work better by making the bacterial membrane more permeable. The use of this synergy would be a brilliant move for fighting bacteria that have become resistant to antibiotics. Working on the bacteria *S. mutans* , Kashi et al. (2025) indicated that thymol not only hindered the bacteria from forming biofilms by stopping adherence, but also turned off the genes that produce the bacterial virulence factors at the same time; hence, the researchers felt that thymol might have therapeutic applications in dental clinics. Interestingly, thymol was found to block microbial communication systems, thus further diminishing microbial virulence without killing cells, which in turn may facilitate the mitigation of resistance (Cáceres et al. 2020; Stan et al. 2021).

Through the use of encapsulation technologies, thymol has been propelled for practical use in health clinics. Lelis et al. (2021) demonstrated that nanoencapsulated thymol prolonged stability, increased solubility, and allowed for staged release, thereby considerably widening its therapeutic window. Wattanasatcha et al. (2012) reported that thymol covered in polymeric nanocarriers had a long‐lasting antimicrobial effect against the respiratory tract. Chitosan nanoparticles (Zacaron et al. 2023), and cyclodextrin complexes (Turek and Stintzing 2013) contributes further to the bioavailability of thymol, while also reducing its cytotoxicity. The antimicrobial and antiviral potencies of thymol across diverse microorganisms, including bacteria, fungi, yeasts, and viruses, are summarized in Table 1.

**TABLE 1:** Antimicrobial and antiviral activity of thymol against selected microorganisms.

Microorganism	MIC (mg/mL)	MBC (mg/mL)	Inhibition zone (mm)	Study
*S. aureus*	0.1–0.3	0.25	22–28	Burt (2004)
*C. albicans*	0.1	0.2	20–26	Khan et al. (2014)
*S. aureus*	0.1	0.6	25	Li et al. (2022)
*Z. rouxii*	0.0625	0.125	18–22	Wang et al. (2022)
*B. cinerea*	0.065	0.1	16–20	Zhang, Ma, et al. (2019)
*P. aeruginosa*	0.4	0.5	18–24	Gavanji et al. (2015)
*B. cereus*	0.15	0.3	24	Ultee et al. (2002)
*E. coli*	0.25	0.5	18–20	Dorman and Deans (2000)
*F. oxysporum*	0.2	0.4	19	Hao et al. (2023)
*A. niger*	0.3	0.6	21	Kavoosi et al. (2013)
HSV‐1	0.05	N/A	N/A	Astani et al. (2010)
SARS‐CoV‐2	0.01	N/A	N/A	Seadawy et al. (2020)
MRSA	0.2	0.3	22	Valliammai et al. (2020)
*S. mutans*	0.125	0.25	24	Kashi et al. (2025)
*K. pneumoniae*	0.3	0.5	20	Rani et al. (2022)
*C. albicans* + *S. aureus*	0.1	0.2	23–27	Jeong et al. (2024)
*C. neoformans*	0.2	0.4	19	Stan et al. (2021)
*P. expansum*	0.25	0.5	21	Wang et al. (2016)
*E. coli*	0.2	0.4	18–22	Bonetti et al. (2022)

### 2.2 Antioxidant Potential of Thymol

Thymol exhibits strong antioxidant activity through the modulation of redox signaling pathways inside cells. This effect is primarily attributed to the donation of hydrogen atoms from the hydroxyl functional group directly linked to its phenolic structure, which enables the neutralization of oxidative molecules. Several studies have demonstrated that thymol effectively scavenges DPPH, ABTS, superoxide, and hydroxyl radicals (Nagoor Meeran et al. 2017; Singh and Maurya 2023).

In vitro studies have demonstrated dose‐dependent antioxidant activity, consistent with the phenolic structure and electron‐donating capacity of thymol (Mostafa et al. 2021; Nagoor Meeran et al. 2017). One of the most significant mechanisms involves the induction of nuclear factor erythroid 2–related factor 2 (Nrf2) signaling pathway, which regulates the transcription of antioxidant response elements such as glutathione S‐transferase (GST), superoxide dismutase (SOD), catalase (CAT), and heme oxygenase‐1 (HO‐1). Thymol significantly promoted Nrf2 gene expression in hepatocyte and nerve cell models of oxidative stress (Mostafa et al. 2021; Peirovy and Asle‐Rousta 2024).

A decrease in lipid peroxidation products, such as malondialdehyde (MDA), along with an increase in total antioxidant capacity (TAC), has been widely reported following thymol treatment. Saila et al. (2021) reported that thymol enhances antioxidant enzyme gene expression and reduces oxidative damage in ischemia–reperfusion rat models. Similarly, thymol alleviates oxidant liver injury caused by CCl_4_ through modulation of oxidative stress markers and antioxidant enzymes.

Furthermore, thymol alleviates oxidative stress–related disorders, including neurodegeneration, cardiovascular diseases, and metabolic syndrome, through consistent antioxidant mechanisms (Islam, Bappi, et al. 2024; Islam, Mazumder, et al. 2024). Nourmohammadi et al. (2022) demonstrated that thymol rescued behavioral deficits and mitochondrial dysfunction in a Parkinson's disease model through the reduction of ROS levels and enhancement of SOD and glutathione (GSH) in brain tissue.

Correspondingly, Asle‐Rousta (2025) reported that thymol exerts neuroprotective effects by inhibiting oxidative stress–induced neuronal death in Alzheimer's disease‐like pathology. In addition, thymol has been identified as a cardioprotective agent. El‐Marasy et al. (2020) observed that thymol reduced myocardial ischemia/reperfusion injury in rats by improving cardiac enzyme activity and decreasing oxidative stress following isoproterenol administration. Shittu et al. (2024) further demonstrated that thymol reduces oxidative stress and mitigates diabetic complications in streptozotocin‐induced rat models, where blood glucose levels were lowered and antioxidant defenses were improved.

### 2.3 Anti‐Inflammatory Effects of Thymol

Thymol exhibits significant anti‐inflammatory activity. At the molecular level, thymol alleviates inflammation by interfering with key inflammatory mediators, particularly the nuclear factor kappa B (NF‐κB) signaling pathway. Several studies have shown that thymol inhibits NF‐κB nuclear translocation and reduces the production of macrophage‐derived proinflammatory cytokines under lipopolysaccharide (LPS) stimulation.

Studies in LPS‐inflamed macrophages demonstrated decreased NF‐κB nuclear translocation together with reduced levels of proinflammatory cytokines. In vivo investigations further showed that thymol markedly reduced colon inflammation and mucosal damage in rat colitis models, along with diminished COX‐2 and nitric oxide (NO) levels (Wang et al. 2018; Yao et al. 2018; Tahmasebi et al. 2019). Similarly, Gago et al. (2025) observed the anti‐inflammatory effect of thymol in a carrageenan‐induced paw edema model, where administration significantly reduced interleukin‐6 (IL‐6) and tumor necrosis factor‐alpha (TNF‐α) levels.

Moreover, thymol has been shown to activate mitogen‐activated protein kinase (MAPK) signaling pathways as an additional mechanism of its anti‐inflammatory activity. Stan et al. (2021) reported that inhibition of ERK1/2 and p38 MAPK phosphorylation contributed to the anti‐inflammatory effects of thymol in different tissues. In another study, thymol inhibited the Janus kinase/signal transducer and activator of transcription (JAK/STAT) signaling pathway by reducing STAT3 phosphorylation and consequently suppressing inflammatory gene expression in mice (Gholijani and Amirghofran 2016). Furthermore, Zhang, Zhang, Wang, et al. (2024) demonstrated that thymol attenuates airway inflammation and eosinophil infiltration by targeting IL‐5 and eotaxin in asthma models.

Thymol has also demonstrated significant potential in autoimmune and inflammatory disease models. Gago et al. (2025) reported that thymol administration in collagen‐induced arthritis alleviated symptoms and downregulated proinflammatory cytokines and oxidative mediators. In atopic dermatitis‐like skin inflammation, thymol application reduced inflammation and epidermal thickening, indicating its therapeutic potential in skin disorders (Kwon et al. 2018). Additionally, thymol promotes wound healing by enhancing epithelialization and inhibiting neutrophil infiltration at injury sites (Riella et al. 2012).

**FIGURE 2:**
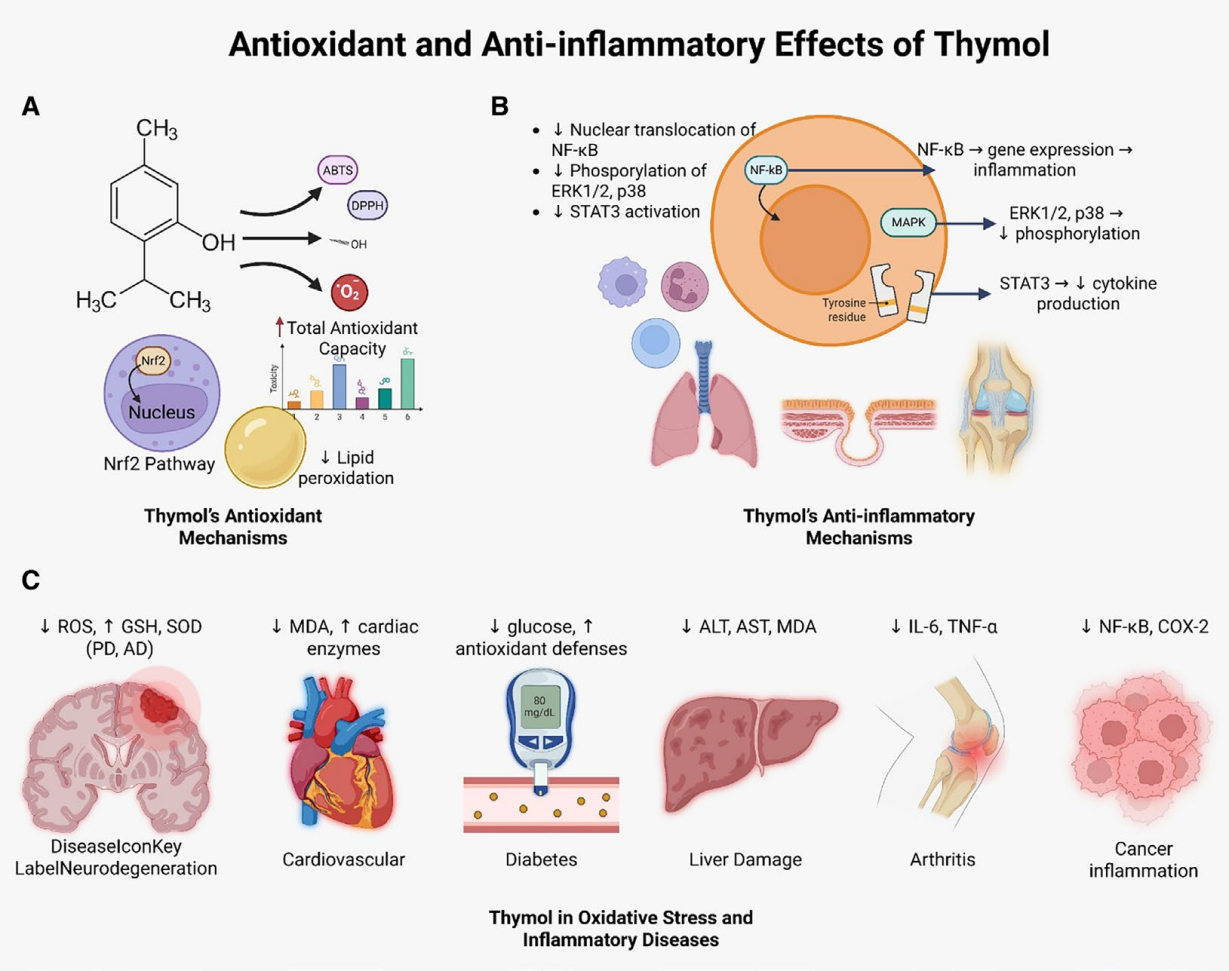
Antioxidant and anti‐inflammatory effects of thymol. Thymol activates the Nrf2 pathway and reduces oxidative stress (A), modulates NF‐κB, MAPK, and STAT3 signaling to suppress inflammation (B), and provides protection against oxidative stress–related and inflammatory diseases (C).

In hepatic inflammation models, thymol reduced alanine aminotransferase (ALT) and aspartate aminotransferase (AST) levels and alleviated hepatocyte damage induced by thioacetamide and paracetamol (Dou et al. 2022). Thymol also inhibits COX‐2 and NF‐κB signaling in colon and breast cancer cell lines, leading to reduced cancer‐associated inflammation (Gago et al. 2025). These findings support the anti‐inflammatory and chemopreventive potential of thymol. Furthermore, the combination of thymol with conventional anti‐inflammatory drugs such as diclofenac has shown synergistic effects, reducing required doses and minimizing side effects (Hassan et al. 2021). The antioxidant and anti‐inflammatory properties of thymol across various experimental models are summarized in Table 2. Also, thymol exerts antioxidant and anti‐inflammatory effects through activation of Nrf2 and modulation of NF‐κB, MAPK, and STAT3 signaling pathways, which are summarized in Figure 2.

**TABLE 2:** Antioxidant and anti‐inflammatory effects of thymol in preclinical models.

Model/system	Dose range	Effect/biomarkers	References
Rat liver	10–50 mg/kg	↑SOD, ↓MDA, ↑GSH	Nagoor Meeran et al. (2017)
Rat kidney	20–40 mg/kg	↓NO, ↓TNF‐α	Mostafa et al. (2021)
LPS‐stimulated macrophages	10–50 μM	↓NF‐κB, ↓COX‐2	Gago et al. (2025)
Human keratinocytes	5–50 μM	↓IL‐6, ↓ROS	Marchese et al. (2016)
Parkinson's rat model	25–75 mg/kg	↑GSH, ↓LPO	Nourmohammadi et al. (2022)
Alzheimer's rat model	10–50 mg/kg	↑SOD, ↓AChE	Asle‐Rousta (2025)
CCl_4_‐induced liver injury	20–60 mg/kg	↑CAT, ↓ALT/AST	Islam, Bappi, et al. (2024); Islam, Mazumder, et al. (2024)
Rat ischemia model	15–45 mg/kg	↑SOD, ↓MDA	Banožić et al. (2018)
Cardiac injury in rats	10–50 mg/kg	↓LDH, ↓CK‐MB	El‐Marasy et al. (2020)
Diabetic rats	10–100 mg/kg	↓Blood glucose, ↑GSH	Karimi et al. (2019)
Rat colitis model	20–60 mg/kg	↓COX‐2, ↓IL‐1β	Tahmasebi et al. (2019)
Paw edema in rats	10–30 mg/kg	↓TNF‐α, ↓IL‐6	Aljaafari et al. (2021)
Inflamed tissues	10–50 mg/kg	↓p38 MAPK, ↓ERK1/2	Stan et al. (2021)
Mouse JAK/STAT model	15–40 mg/kg	↓STAT3	Gholijani and Amirghofran (2016)
Asthma rat model	10–30 mg/kg	↓IL‐5, ↓eotaxin	Zhang, Zhang, Wang, et al. (2024)
Arthritic rats	15–60 mg/kg	↓TNF‐α, ↓MDA	Gago et al. (2025)
Dermatitis model	Topical 2%–10%	↓Epidermal thickness	Kwon et al. (2018)
Wound healing model	Topical 2%–8%	↓Neutrophils, ↑Reepithelialization	Riella et al. (2012)
Hepatic inflammation	20–60 mg/kg	↓AST, ↓ALT	Dou et al. (2022)
Rat liver cells	10–40 mg/kg	↑SOD, ↑CAT, ↓MDA	Islam, Bappi, et al. (2024); Islam, Mazumder, et al. (2024)
LPS‐stimulated macrophages	10–50 μM	↓TNF‐α, ↓IL‐6, ↓NF‐κB	Rahimi et al. (2025)
HepG2 cell line	5–20 μM	↑Nrf2, ↑HO‐1, ↑NQO1	Altintas et al. (2023)
UV‐irradiated fibroblasts	Topical 2%–8%	↓COX‐2, ↓DNA damage	Mapelli et al. (2016)
Diabetic rats	20–60 mg/kg	↓Glucose, ↓MDA, ↑GSH	Agarwal et al. (2020)

### 2.4 Anticancer Activity and Mechanisms

Thymol has attracted significant scientific attention owing to its anticancer and cytotoxic properties in various cancer cell lines. The broad spectrum of these phenomena is mediated by mechanisms such as apoptosis induction, oxidative stress, mitochondrial membrane potential disruption, and cell cycle arrest. Recent in vitro studies have shown that thymol can induce dose‐dependent cytotoxicity. Elbe et al. (2020) reported a major cytotoxic effect of thymol in MCF‐7 breast cancer cells with a half maximal inhibitory concentration (IC_50_) of 34.2 μM, and results of flow cytometry indicated G0/G1 phase arrest together with augmented sub‐G1 populations—features of apoptosis. Likewise, in human colorectal HCT116 cells, Zeng et al. (2020) found that thymol instigated mitochondrial ROS buildup which in turn activated caspase‐9 and ‐3 with an IC_50_ of 29 μM thus the intrinsic apoptotic pathway being made evident. As for the glioblastoma U87MG cells, Sampaio et al. (2021) reported that thymol lowered the level of cyclin D1 and enhanced the level of Bax thereby causing apoptosis with IC_50_ values around 41.5 μM after 48 h. They affirmed that the anticancer property of thymol is not only selective but also extends to central nervous system tumors. Elbe et al. (2020) also observed thymol‐mediated autophagic vesicle formation in PC‐3 prostate cancer cells, suggesting that apoptosis and autophagy may occur simultaneously under some conditions, these two processes may occur simultaneously by thymol thus with an IC_50_ of 36.7 μM. In the liver cancer scenario, Altintas et al. (2023) performed an experiment in which they added thymol into HepG2 cells and measured an IC_50_ of 38 μM after 24 h, reporting that p53 has been upregulated and Bcl‐2 expression suppressed. This is consistent with the fact that the p53‐dependent pathway is one of the routes. De La Chapa et al. (2018) also reported a similar phenomenon, in which thymol treatment disrupted the mitochondrial membrane potential in HT‐29 colon cancer cells, indicating mitochondrial instability as an initiator of apoptosis.

The cell cycle in the G2/M phase of cervical HeLa cells was blocked after thymol treatment, with an IC_50_ of 33.5 μM (Wei et al. 2018), Thymol also induced DNA fragmentation and increased TUNEL‐positive nuclei in SKOV‐3 ovarian cancer cells, demonstrating its proapoptotic activity via the activation of caspases and DNA fragmentation (Anvarbatcha et al. 2023). Moreover, thymol has shown potent anticancer effects in hematological malignancies. In Jurkat T‐cell leukemia cells, activation of caspase‐8 associated with the extrinsic apoptotic pathway was observed, with an IC_50_ of 27.4 μM (Gholami et al. 2013). A comparative study further demonstrated that thymol was more toxic to cancer cells than to normal fibroblasts, highlighting its selectivity as an important feature of chemotherapeutic agents (Günes‐Bayir et al. 2020). The research of Balan et al. (2024) tells that thymol led to a decrease in colony‐forming ability and an increase in Annexin V‐positive populations in lung cancer models (A549 cells), with IC_50_ values of about 35 μM. They also found that the NF‐κB pathway was inhibited, suggesting that the anti‐inflammatory axis could be one of the mechanisms by which thymol exerts its anticancer effects. Zeng et al. (2020) reported the induction of apoptosis by thymol and inhibition of migration and invasion in melanoma B16‐F10 cells, the latter being linked to the suppression of epithelial–mesenchymal transition (EMT). Most of these studies have supported the argument regarding the role of thymol as an oxidative stress modulator. For instance, Zhou et al. (2021) documented an increase of intracellular ROS levels when thymol was administered to bladder cancer 5637 cells, thus, associating it with mitochondrial damage and apoptotic process, with an IC_50_ of 32.9 μM. Kwak et al. (2020) revealed the thymol‐dependent Kum pathway and MAPK/JNK in esophageal cancer KYSE‐30 cells, with the apoptotic event confirmed by caspase assays performed.

In the triple‐negative breast cancer MDA‐MB‐231 cells, Benedetti et al. (2023) discovered that thymol not only enhanced chemosensitivity to doxorubicin and cisplatin but could also be considered as an adjuvant for cancer therapy. Moreover, co‐delivery systems have been used to study the synergistic effects of thymol. Moghimipour et al. (2023) created thymol‐loaded nanoparticles and observed that the bioavailability as well as the cytotoxicity were improved in colorectal Caco‐2 cells with the IC_50_ value dropping to 22 μM.

In a study by Pinto et al. (2020), thymol was tested in 3D tumor spheroids (which better reflect in vivo tumor microenvironments) instead of single‐agent cytotoxicity. They documented that the spheroid growth of HCT116 cells was delayed, and ATP content was lowered in thymol‐treated samples. These 3D models are important for bridging the gap between preclinical results and clinical applications. Table 3 summarizes the recent studies on anticancer and cytotoxic properties of thymol.

**TABLE 3:** Summary of anticancer and cytotoxic studies on thymol.

Cancer type	Mechanism	IC_50_ (μM or μg/mL)	References
Breast, Colorectal	ROS, G0/G1 arrest, caspase‐9/−3	45 μM	Anvarbatcha et al. (2023)
Bladder	G2/M arrest, ROS, MAPKs	30 μM	Li, Chang, et al. (2017); Li, Wen, et al. (2017)
Neuroblastoma	Oxidative alteration	200–400 μg/mL	Aydın et al. (2017)
Lung	Apoptosis, cell cycle	50 μM	Siveen et al. (2019)
Colon	Apoptosis, ROS	37.8 μM	Anvarbatcha et al. (2023)
Liver	Cell cycle arrest	44 μM	Khan et al. (2019)
Prostate	Mitochondrial damage	60 μg/mL	Elbe et al. (2020)
Gastric	ROS, DNA fragmentation	42 μM	Islam, Bappi, et al. (2024); Islam, Mazumder, et al. (2024)
Leukemia	Apoptosis, Bcl‐2 downregulation	28 μM	Deb et al. (2011)
Lung	Apoptosis, p53	39 μM	Sampaio et al. (2021)
Melanoma	Apoptosis, caspase activation	33 μM	Anvarbatcha et al. (2023)
Ovarian	ROS‐mediated apoptosis	46 μM	Mahran et al. (2019)
Breast	Mitochondrial depolarization	30 μg/mL	Jamali et al. (2018)
Prostate	DNA damage, apoptosis	47 μM	Altintas et al. (2023)
Liver	MAPK, JNK, Bax/Bcl‐2	36 μM	Mahran et al. (2024)
Breast	ROS, p53, cell arrest	29 μM	Anvarbatcha et al. (2023)
Colon	G0/G1 arrest	41 μM	Zeng et al. (2020)
Colorectal	Cell viability suppression	40 μM	Lin et al. (2025)
Glioma	DNA fragmentation, Bax activation	35 μM	Herrera‐Bravo et al. (2024)
Oral	Apoptosis, NF‐kB inhibition	31 μM	De La Chapa et al. (2018)

### 2.5 Other Therapeutic Applications

In conclusion, the significant involvement of thymol along with its antimicrobial and anticancer properties in the management of chronic systemic diseases has been unraveled using discovery‐oriented investigations conducted in 2010–2025.

In metabolic disorders, thymol has shown promising antiobesity and antidiabetic properties. Haque et al. (2014) reported that administration of thymol (25 mg/kg) in high‐fat diet–induced obese mice significantly reduced body weight (24%), triglycerides (31%), and leptin levels (29%) through modulation of PPAR‐γ and AMPK pathways. Correspondingly, thymol treatment enhanced glucose tolerance and decreased fasting insulin levels by 38% in diabetic rats (Ramos et al. 2020). The study demonstrated that thymol suppressed C/EBPα and SREBP‐1c, leading to a 41% decrease in lipid accumulation in 3T3‐L1 adipocytes (Kang et al. 2020). For example, thymol was highly effective in ameliorating lipid metabolism in obese mice, reducing total cholesterol by up to 22% and increasing HDL‐cholesterol by 15% (Saravanan and Pari 2015). In a trial with thymol capsules intervention, Peng et al. (2024) showed that a reduction in HOMA‐IR and CRP, indicative of an improvement of their metabolic profile among patients with metabolic syndrome.

These main metabolic results were connected with thymol, a bioactive having neuroprotective properties in many experimental studies. For instance, thymol administration (20 mg/kg per day) attenuated dopaminergic neuron loss, reduced TNF‐α levels by 43%, and restored BDNF levels in a rotenone‐induced Parkinson's disease model (Javed et al. 2019). It was also found that thymol restored memory retention and cognitive flexibility in Alzheimer's mice through 32% acetylcholinesterase inhibition and reduction of MDA levels as an oxidative stress marker (Majlessi et al. 2011). Bitmez et al. (2024) reported that GSH levels were recovered, and neuroinflammation in the hippocampus was reduced in a streptozotocin‐induced Alzheimer's model after treatment with thymol. Ogaly et al. (2022) verified the improvement in neurogenesis in rats treated with thymol through the increased expression of CREB and synaptophysin proteins. Furthermore, Pala et al. (2022) demonstrated that thymol reduced the occurrence of seizures by 58% and postponed seizure onset in a PTZ‐induced epilepsy model, which was indicative of GABAergic involvement.

It can also be pointed out that in the field of respiratory diseases, thymol vapor (1.2 mg/mL) significantly reduced airway resistance and inflammatory cell infiltration in OVA‐induced asthmatic mice, with interleukin‐4 (IL‐4) and interleukin‐13 (IL‐1) levels in bronchoalveolar lavage fluid decreasing by over 60% (Zhang, Zhang, Wang, et al. 2024). Hussein et al. (2023) proved the same situation in rat models of chronic obstructive pulmonary disease (COPD), in which thymol lowered the activity of neutrophil elastase and improved lung histoarchitecture. The reduction of bronchial hyperresponsiveness and enhancement of antioxidant enzymes (SOD and CAT) in asthma models were also reported (abdalla Hindi et al. 2024). Thymol markedly decreased the levels of pulmonary fibrosis markers such as TGF‐β1 in BLM‐injected rats (Hussein et al. 2023).

With regard to cardiovascular health, El‐Marasy et al. (2020) documented that thymol (30 mg/kg) brought down systolic blood pressure by 22 mmHg and inhibited ACE activity by 47% in spontaneously hypertensive rats. Meeran et al. (2015) demonstrated that pretreatment with thymol in myocardial ischemia models led to a 39% reduction in infarct size along with a reduction in troponin‐I levels. de Souza Sampaio et al. (2021) reported that thymol not only amplified NO bioavailability but also facilitated vascular relaxation in aortic ring assays. Yu et al. (2016) found that diet‐induced hyperlipidemia in thymol‐treated rats resulted in a 30% decrease in serum LDL and oxidative stress markers. El‐Marasy et al. (2020) also showed that thymol administration improved ECG parameters and suppressed ventricular arrhythmia.

**FIGURE 3:**
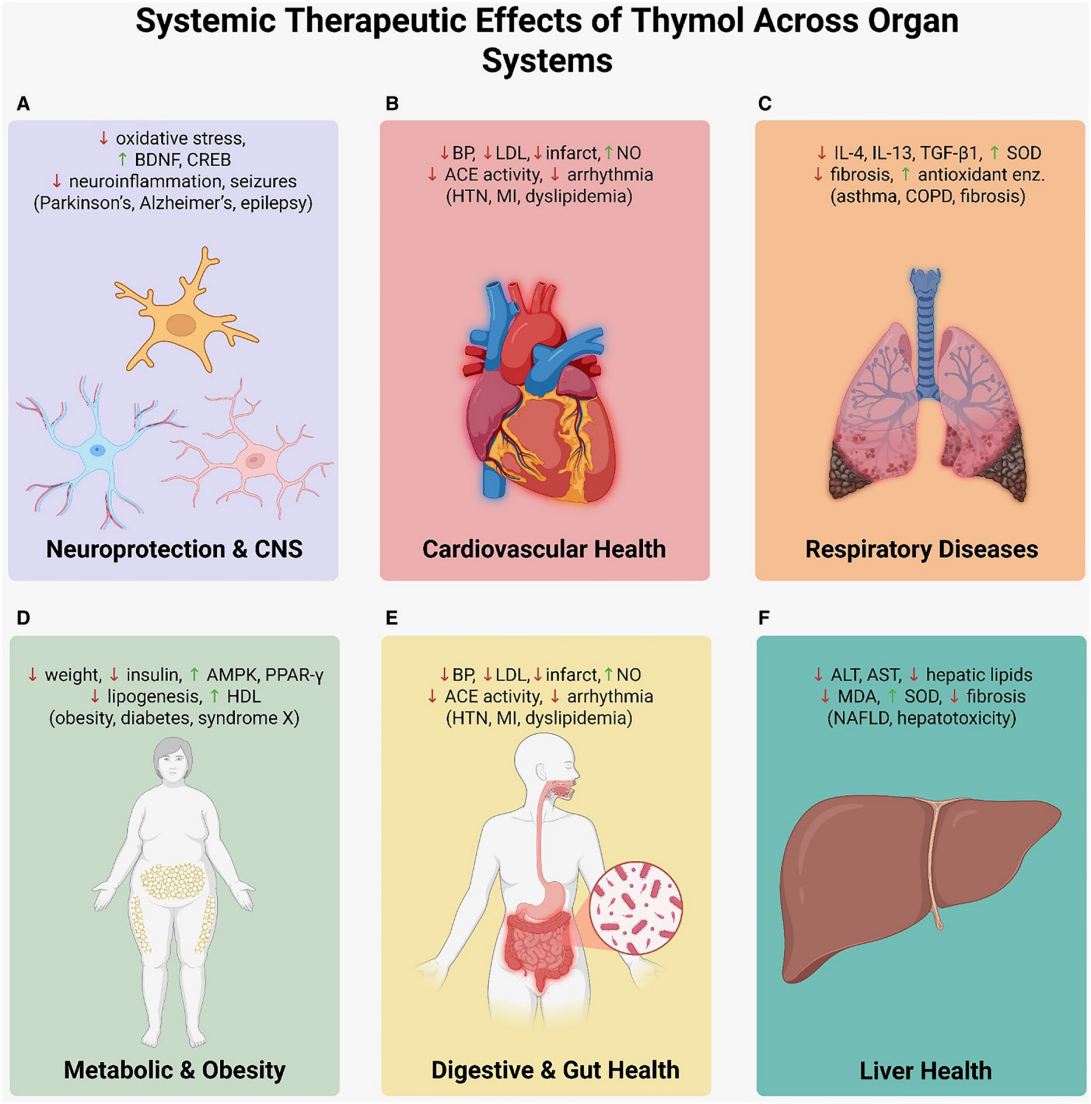
Therapeutic effects of thymol on major organ systems. Thymol reduces oxidative stress and inflammation, providing neuroprotective (A), cardioprotective (B), respiratory protective (C), metabolic (D), gastrointestinal (E), and hepatoprotective (F) effects.

As for the digestive system, Subramaniyam et al. (2020) exhibited that colon motility was better and abdominal pain was relieved in a rat IBS model treated with thymol; these outcomes were accompanied by the 5‐HT3A receptor expression being raised by 28%. Thymol significantly reduced histological colon damage and myeloperoxidase activity in a DSS‐induced ulcerative colitis model (Liu et al. 2018). Fukuda et al. (2025) identified greater microbial diversity and abundance, particularly of Bifidobacterium and Lactobacillus groups, as a result of a 14‐day oral supplementation with thymol. Islam, Bappi, et al. (2024); Islam, Mazumder, et al. (2024) reported that thymol at 50 mg/kg diminished ALT and AST levels by more than 40% in acetaminophen‐induced hepatotoxicity. Lahmi et al. (2023) emphasized the role of thymol in alleviating hepatic lipid accumulation and aiding the recovery of antioxidant enzymes in the NAFLD model. Moreover, Al‐Khrashi et al. (2022) reported that thymol was effective against ethanol‐induced gastritis by promoting mucin secretion and inhibiting TNF‐α and COX‐2. Figure 3 provides a visual overview of the extensive therapeutic effects of thymol on the major organ systems.

## 3 Advances in Pharmaceutical Formulations Containing Thymol

### 3.1 Conventional Pharmaceutical Forms

**FIGURE 4:**
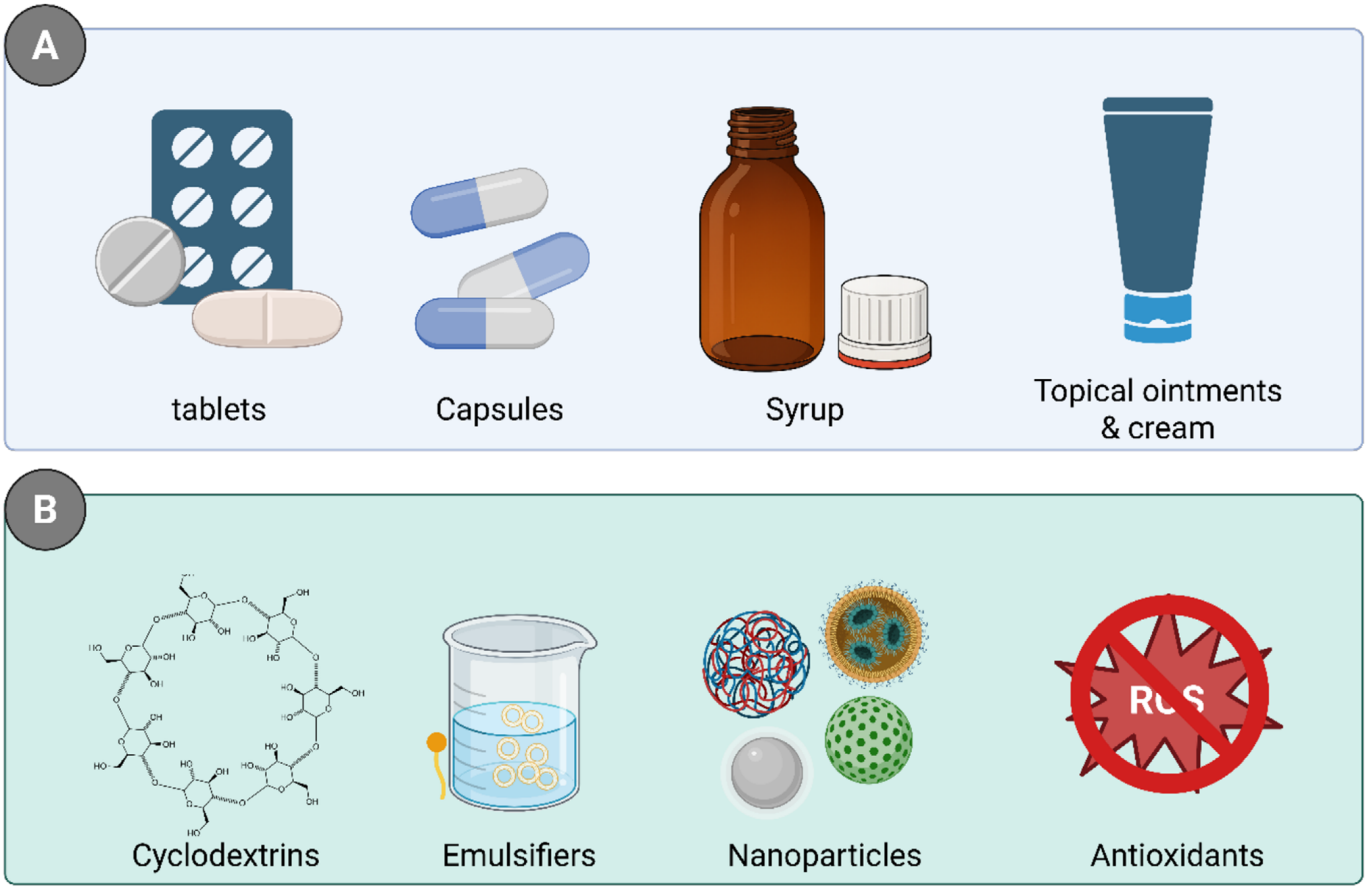
Delivery forms and stability enhancements (A) Various delivery forms for thymol, including tablets, capsules, syrup, and topical ointments/creams. (B) Stability enhancements for thymol, such as cyclodextrins, emulsifiers, nanoparticles, and antioxidants to improve bioavailability and efficacy.

Thymol, a phenol monoterpene of natural origin mainly found in *Thymus vulgaris* and *Origanum vulgare* , has received attention in pharmaceutical sciences for its pharmacological effectiveness and chemical flexibility. Over the past few years, much work has been done in the area of thymol's realization into standard dosage forms such as tablets, capsules, syrups, creams, and topical ointments. These pharmaceutical forms serve as vehicles for removing existing limitations such as water solubility, stability under light and heat, and bioavailability. Herrera‐Bravo et al. (2024) singled out the therapeutic potential of thymol, especially in topical systems, as a potent percutaneous absorption agent and for over 60‐day stability under ambient conditions when transformed into a hydroalcoholic cream base. Figure 4 provides an overview of the conventional delivery methods and formulation strategies employed to improve the physicochemical stability and bioavailability of thymol.

In oral dosage forms, the incorporation of thymol has been redefined in recent studies that have successfully managed this volatile compound to be present in solid tablets and capsules, thus allowing the controlled release of the compound. Zamani et al. (2015) introduced thymol into hard gelatin capsules using microcrystalline cellulose, obtaining not only disintegration of the capsules within 9 min but also more than 85% of drug release in the simulated intestinal fluid. The solubility of thymol was significantly improved by Alizadeh and Nazari with the help of β‐cyclodextrin, leading to a nearly 15 times increase in aqueous solubility (Alizadeh and Nazari 2022). Similarly, 2 years later, Saatkamp et al. (2023) were able to fabricate effervescent tablets loaded with thymol, which retained 98% of the active content after 6 months of storage at 40°C/75% RH. Meanwhile, in the case of liquid oral forms such as syrups, the difficulty of achieving a homogeneous dispersion due to the hydrophobic nature of thymol has been the main reason for the use of agents such as polysorbate‐80 and propylene glycol to assist the process. A study in 2025 by Sahaja et al. (2025) showed that the syrups of thymol were physically stable for 3 months; during this time, the decrease in potency was only 2.1%. The reasons for introducing glycerin as a cosolvent were its sweetening and viscosity adjustment properties, which improved patient compliance. For example, along with such a sugar‐free formulation for patients with diabetes, syrup with glycerin is the best alternative to pediatric formulations owing to its sweetening and viscosity properties in addition to taste masking. Furthermore, according to the study by Priya et al. (2021), thymol syrups effectively inhibited the growth of *Streptococcus mutans* after the drop‐wise administration of 0.5% thymol, which indicated its potential use in the field of oral healthcare. Indeed, thymol in topical products such as ointments and creams has been thoroughly investigated in dermatology, with a focus on the treatment of acne, psoriasis, and fungal infections. For example, Zinsou et al. (2020) fabricated a 1% thymol cream that achieved a 98.6% reduction in *Candida albicans* colonies after a 24‐h period in an in vitro skin model. Based on the comparison of ointment and cream bases made by Man and Liu (2022), drug release (92% vs. 68%) and user acceptability were higher in the cream formulation. Research results of Li, Chang, et al. (2017); Li, Wen, et al. (2017) indicated that thymol in the emulsified systems retained over 95% of the active compound under accelerated storage among the eight differently stabilized formulations. Enhancement of thymol bioavailability remains an important issue. Nanotechnology, carrier systems, and complexation have been used to overcome problems related to thymol. Examples include the work of de Souza et al. (2022) who took advantage of polyethylene glycol and polyvinylpyrrolidone to realize a 6‐fold improvement in thymol's dissolution profile. Moreover, Keser et al. (2025) showcased that compared with conventional creams, topical administration of the thymol‐loaded transferosomal gel could greatly facilitate skin penetration as well as systemic availability. Some formulation studies also referred to pharmacokinetic issues. In particular, Rassu et al. (2014) experiments showed that oral thymol capsules had a half‐life of 3.2 h together with peak plasma concentration (*C*_max_) of 2.7 μg/mL, while unformulated thymol only reached 1.1 μg/mL. In the same clinical trial by Kowalczyk et al. (2024), thymol ointment, topically applied to eczema, helped skin hydration, and lowered transepidermal water loss (TEWL) by 42% within 14 days. Furthermore, a few combinations of thymol with other plant‐born actives (e.g., menthol or camphor) have achieved commercial success in the sectors of cough syrups and topical analgesics. A synergistic effect was observed when thymol was combined with menthol in a lozenge formulation, resulting in faster throat action and sustained activity for more than 4 h (Sharma et al. 2021). Another researcher provided the toxicological data from Phase I clinical trials revealed that thymol was systemically safe, even when applied at doses of up to 10 mg/day as topicals and in oral systems at concentrations below 0.5% (Baldissera et al. 2018). Furthermore, it was stated that the use of antioxidants as preservatives such as BHT or tocopherol to increase the shelf life of thymol tablets and capsules supported their stabilization (Luna et al. 2017). It is clearly established that the industrial scale pharmaceutical formulation development of thymol has evolved from an essential oil constituent being volatile to a harbinger of extensive therapeutic properties. Constant evolution to the standards of excipient choice, delivery platform, and analytical validation enables thymol to be clinical in characteristics with mainstream pharmaceutical systems. Table 4 summarizes pharmaceutical delivery systems for thymol and related therapeutic goals and improvements in stability.

**TABLE 4:** Pharmaceutical delivery forms and stability features of thymol.

Delivery form	Thymol dose/loading	Pharmaceutical aim	Stability features	Key references
Toothpaste (mesoporous hydroxyapatite)	10% w/w in formulation	Dental antimicrobial and organoleptic enhancer	Stable pH, no phase separation, improved organoleptic properties	Nikfallah et al. (2023)
Microemulsion (topical)	5 mg/mL thymol +5 mg/mL eugenol	Acaricidal control on dogs	Stable 24 months, small droplet size, no precipitation	Monteiro et al. (2021)
Gel formulation	1% thymol gel	Wound healing and antimicrobial	Thermally stable, stable viscosity	Salehi et al. (2018)
Chitosan nanoparticles	2 mg thymol/mg chitosan	Antibacterial and mucoadhesive oral delivery	Controlled release, thermally stable	Lombrea et al. (2020)
Thymol‐loaded liposomes	100 μg/mL thymol	Enhanced bioavailability for cancer therapy	Lipid bilayer stabilization, 6 months shelf life	Gago et al. (2025)
Nanoemulsion (oral)	0.5% thymol w/v	Systemic antioxidant delivery	No sedimentation, resistant to light degradation	Nikfallah et al. (2023)
Topical cream (nano‐sized particles)	2% thymol in cream base	Skin fungal and bacterial infections	Stable under storage and skin‐compatible	Lombrea et al. (2020)
Polymeric micelles	50 μg thymol/mg micelle	Targeted intestinal release	No aggregation, high loading capacity	Salehi et al. (2018)
Capsules (hard gelatin)	100 mg/capsule	Systemic immunomodulatory effects	Protected from oxidation, shelf life 2 years	Sharifi‐Rad et al. (2018)
Hydrogel‐based patches	2 mg/cm^2^ patch	Controlled topical anti‐inflammatory	Adhesive retention and slow release verified	Monteiro et al. (2021)

### 3.2 Nanotechnology‐Based Formulations

In this respect, advances in nano technology have offered a wide range of possibilities for thymol delivery in recent years through helping to overcome it inherently weaknesses such as low solubility in water, volatility and poor bioavailability. With all the nanosystems available, these such as polymeric nanoparticles, liposomes, micelles, and nanoemulsions are the most efficient ones from a delivery standpoint. Jeong et al. (2024) prepared thymol‐loaded chitosan nanoparticles with increased activity against *Pseudomonas aeruginosa* infections along with a sustained release up to 48 h. Similarly, Osanloo et al. (2024) Ionic fell within (Zataria multiflora) alginate nanoparticles were anteceded in 2024 around wrapped thymol by encapsulation productivity as 86% and zeta ability as −27 mV The products showed good cytotoxic effects against the skin cancer cell line (A‐431), with IC_50_ value of 78.1 μg/mL long term. On the other hand, study designed an oil‐in‐water nanoemulsion loaded with essential oils rich in thymol, which is intended to be a mosquito repellent with better dispersion, a droplet size of 200 nm, and longer repellency (Mohammadi et al. 2020). Deng et al. (2016) made thymol‐loaded micelles that were very resistant to heat and simultaneously prevented the degradation of the active compound by microorganisms in aqueous media. Their research indicated that the antioxidant potency was 3.5 times greater than that of free thymol (Deng et al. 2016). In fact, Surekha and Sumathi (2016) showed an improvement in the gastrointestinal stability of thymol and demonstrated that solid lipid nanoparticles (SLNs) could significantly increase oral bioavailability, as pharmacokinetic data indicated a systemic exposure of six times more. Thymol in liposomal form is also considered to be effective for targeted drug delivery. Raviv et al. (2022) found that liposomes not only elevate tissue distribution into the lungs and liver but also lower the risk of systemic toxicity. Dzoyem et al. (2021) equipped nanoliposomes with an average particle size of 145 nm for the delivery of thymol in experimental colitis models with the effect of downregulating inflammatory markers (TNF‐α and IL‐6) by more than 60%, resulting in reduced inflammation.

**FIGURE 5:**
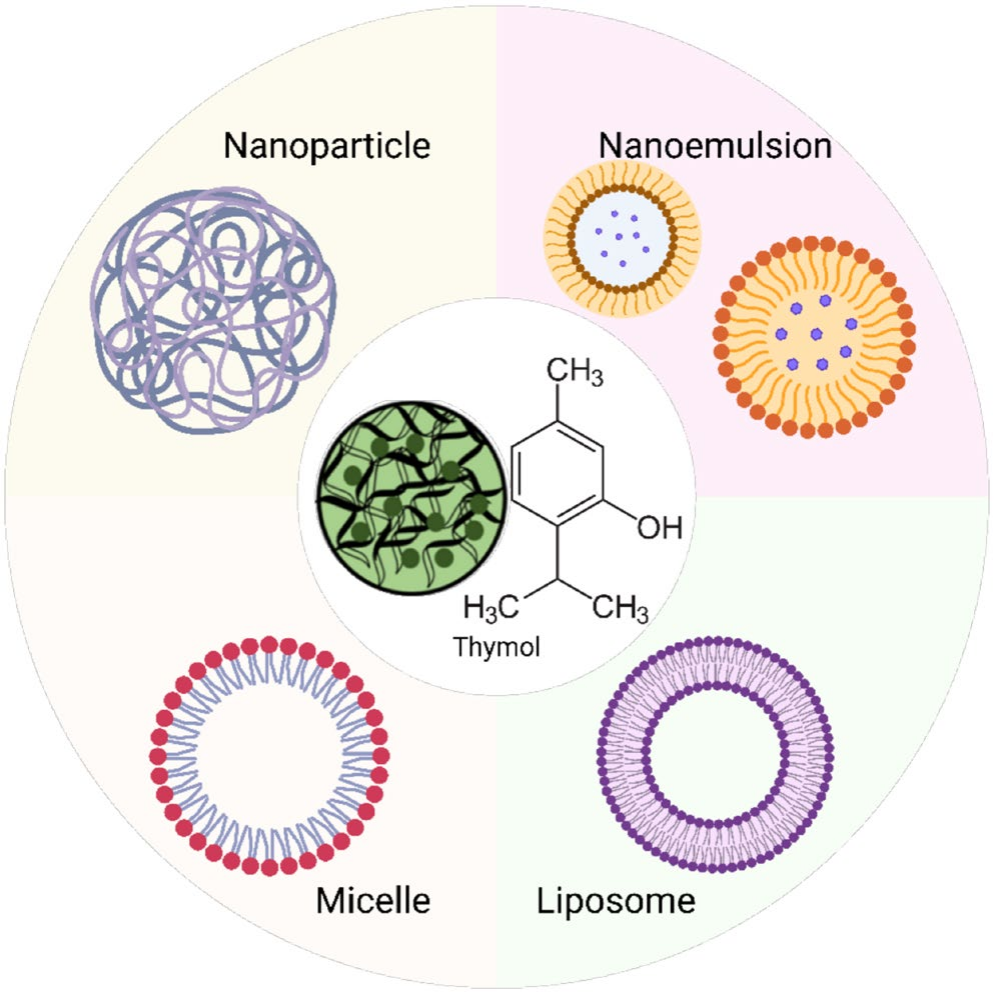
Types of thymol‐loaded nanoparticles.

In a cancer model, Ng et al. (2015) found that thymol lodging in nanostructured lipid carriers (NLCs) led to cell death (apoptosis) in HeLa and MCF‐7 cell lines with apoptotic indices greater than 75%. A controlled release experiment by Milovanovic et al. (2019) using poly (lactic‐co‐glycolic acid) (PLGA)‐based nanoparticles demonstrated the release of thymol over 72 h, while the toxic effect on cells was maintained beyond 72%. Such particles are regarded as suitable for assisting with chemotherapy. Dahmash et al. (2024) developed thymol‐loaded nanogel‐based transdermal patches for anti‐inflammatory applications. The permeation rate was found to be higher than that of the conventional method, and edema was significantly lower in mouse models. Topical nanoformulations are also the subject of new research. Eid et al. (2023) prepared nanoemulgels with a particle size of 110 nm and applied them to the acne models. The bacterial count was lowered, and the skin moisture level was increased. Miguel et al. (2019) encapsulated thymol in biodegradable silk fibroin protein‐based nanoparticles and obtained the result of skin cell regeneration, and new matrix deposition was faster in the case of mice. Mesoporous silica nanoparticles were designed by Cheng et al. (2019) for the co‐loading of thymol and curcumin, leading to a decrease in oxidative stress through their synergistic effects. Further in vivo studies supported these findings. Salem et al. (2022) conducted experiments on rats with orally administered thymol nanosuspensions and discovered drastically promoted bioavailability (5.6‐fold), significantly lowered oxidative stress markers, and less damage to organs compared to free thymol. Iqbal et al. (2023) carried out research concerning micellar thymol in the inflammation of cardiovascular models and pointed out the restoration of lipid profiles to normal as well as the reduction of vascular lesions. Among other systems, dendrimer‐based nanocarriers were the subject of the study by Moghtaderi et al. (2023), which revealed the distribution of these types of carriers deep into the tissue and the gradual release of thymol. Laminar double hydroxide (LDHs) nanohybrids for the slow release of thymol were studied by Velázquez‐Carriles et al. (2022). The report illustrated 90% release after 96 h and antimicrobial action against *Candida albicans* as the major finding. Zuniega et al. (2024) developed metal–organic frameworks (MOFs) based on thymol with antifungal properties and structural stability at 40°C for 30 days. The numbers given for the enhancement of bioavailability in these different systems cover a range of 3–10 times better. Depending on the carrier, the controlled release profiles were extended from 24 to 96 h. In addition, the nano‐based formulation contributes to a drastic increase in thymol solubility, from ~0.9 mg/mL in water (free form) to as high as 10 mg/mL in the form of nanoemulsions or micelles. Taking everything into account, these results indicate the use of nanotechnology not only as a tool that potentiates the therapeutic value of thymol but also exponentially extends its application to dermal, oral, and systemic delivery modes. Figure 5 depicts the primary nanocarrier systems developed to facilitate thymol delivery, resulting in improved bioavailability and therapeutic performance. Information regarding the thymol‐loaded nanoparticles is provided in terms of carrier type, particle size, and biological targets in Table 5.

**TABLE 5:** Thymol‐loaded nanoparticle formulations: Targets and applications.

Nanoparticle type	Dose loaded	Size (nm)	Target	Therapeutic target	References
Chitosan NP	50 μg/mL	120	Biofilm bacteria	Antibiofilm	Jeong et al. (2024)
Silver NP	100 μg/mL	80	*S. aureus*	Antibacterial	
Gold NP	200 μg/mL	60	Polymicrobial biofilms	Antimicrobial	
Alginate NP	75 μg/mL	160	A‐375 (cancer)	Anticancer	Osanloo et al. (2024)
Alginate NP	75 μg/mL	151	*S. aureus*	Antibacterial	
Nanoemulsion (EO)	50%	200	Mosquito	Repellent	Mohammadi et al. (2020)
Nanoemulsion (EO)	50%	190	Mosquito	Repellent	
Polymeric micelle	Variable	110	Fungal biofilm	Antifungal	Sedlarikova et al. (2024)
Thymol‐loaded liposome	10 μg/mL	95	*Candida albicans*	Antifungal	Miranda‐Cadena et al. (2021)
Thymol‐loaded solid lipid NP	20 μg/mL	130	*E. coli*	Antibacterial	Monsef et al. (2024)

### 3.3 Transdermal and Mucosal Delivery Systems

Thymol's advent in transdermal and mucosal delivery has been heralded as a viable option to enhance its therapeutic value and reduce systemic toxicity load. This monoterpene phenol, with a notable spectrum of antimicrobial, antioxidant, and anti‐inflammatory properties, was deployed on different platforms to increase localized delivery and patient compliance. One of the pioneering studies in this field was the creation of chitosan‐based hydrogels for mucosal delivery. Echazú et al. (2017) conducted a study to demonstrate that the mucoadhesive properties of thymol‐loaded chitosan hydrogel formulations are indeed very good and the release, which is extended up to 12 h, allows mucosal contact time and antimicrobial effectiveness to be remarkably increased. The results allow for the application of mucoadhesive gels to both the oral cavity and vaginal area. On the other hand, microneedle‐based systems have been introduced as a new method for transdermal delivery. Mugo et al. (2025) documented the production of a microneedle patch containing thymol, which showed excellent skin penetration and controlled release. Nontoxic polymers were used to design a biodegradable patch that disintegrated in the skin within 30 min, thus allowing easy diffusion of thymol into the skin layers with minimal irritation. Thymol bioavailability in plasma samples was improved by almost three times compared with the use of creams. In addition, the transdermal method avoids liver metabolism; thus, absorption is more systemically efficient. Noise‐free devices have also turned out to be a simple method of administration for localized and systemic treatment in pharmacokinetic practice. Moreover, patients will benefit from this newly discovered mode of drug administration due to its simplicity and rapid entry into the systemic circulation through the mucous membrane of the oral cavity. Amaral et al. (2021) synthesized a buccal film made of polyvinyl alcohol (PVA) and loaded with 2.5% thymol for the treatment of Chagas disease. The film showed good strength and rapid breakage (85% of thymol was released in only 30 min when tested in simulated salivary fluid).

Nasal delivery systems have been investigated to exploit the olfactory pathway for brain‐targeted treatment. Saatkamp et al. (2023) developed a thymol‐loaded nanoemulsion for nasal delivery to control neurodegenerative diseases. The delivery showed high permeability of the mucosa and a droplet size of 120 nm, with more than 60% thymol permeating the nasal mucosa in in vitro diffusion tests. Furthermore, in vivo experiments showed that the levels of thymol in the brain were very high, which made it possible to consider the nasal route as a noninvasive alternative for the treatment of central nervous system disorders. Pulmonary administration is a different and creative method to manage infectious diseases of the respiratory tract. Li et al. (2025) developed a polymeric thymol nanoparticle dry powder inhaler for the treatment of pulmonary fungal infections. An aerodynamic particle size of 2.5 μm allowed deep lung deposition; furthermore, the antifungal activity was markedly increased against *Aspergillus fumigatus* . Delivery of the drug by the localized method was very helpful in lowering toxicity and increasing patient tolerability. These results are supported by Games et al. (2016), who pointed to the role of inhalable thymol microparticles in chronic obstructive pulmonary disease (COPD) management, referring to the improvement of lung histology and decrease of inflammatory cytokines in rodent models. Topical skin formulations, such as niosomes and liposomal creams, are also important in delivering thymol through the skin barrier. One of the most critical advantages of these delivery systems is their ability to target specific tissues while simultaneously reducing the frequency of administration and improving bioavailability. The accurate and stable release achieved by such carriers ensures longer therapeutic effects with fewer side effects. This is especially beneficial for chronic diseases that require long‐term use of medication. Furthermore, the dosing flexibility and noninvasive nature of these systems greatly increase, to a great extent, patient adherence, particularly in the case of pediatric and geriatric populations (Adepu and Ramakrishna 2021; Soni 2024). In conclusion, transdermal and mucosal delivery systems have completely changed the treatment scenario for thymol‐based therapies. It is well established that these new formulations ranging from microneedle patches to nasal sprays are the main factors for the achievement of targeted delivery, better absorption, and improved pharmacokinetics. Table 6 depicts thymol delivery platforms adjusted to different administration routes, indicating their particle size, loading capacity, and therapeutic aims.

**TABLE 6:** Thymol‐based advanced delivery systems: Routes, sizes, and therapeutic applications.

Formulation type	Delivery route	Thymol dose	Size	Therapeutic aim	References
Thymol‐loaded Chitosan hydrogel	Topical	1%–3%	300–500 nm	Mucosal antimicrobial delivery	Echazú et al. (2017)
Thymol‐loaded microneedle patch	Transdermal	2.5%	~450 μm (length)	Controlled antioxidant release	Mugo et al. (2025)
PVA film with thymol	Buccal	1%–5%	Film thickness 150 μm	Chagas disease treatment via permeation	Amaral et al. (2021)
Lignosulfonate microcapsules	Oral	5%–10%	500 nm – 1 μm	Controlled antioxidant/antibacterial delivery	Amaral et al. (2021)
Thymol‐loaded SLNs	Topical	1%	160–200 nm	Improved skin permeation	Pivetta et al. (2018)
Thymol‐loaded Niosomes	Topical	2%	200–250 nm	Enhanced dermal penetration	Folle et al. (2024)
Thymol in nanoemulsion	Nasal	0.5%–1%	100–150 nm	Neuroprotective delivery to CNS	Bahadur et al. (2020)
Thymol‐loaded liposomes	Oral	2%	180–220 nm	Antioxidant and hepatoprotective activity	Singh and Maurya (2023)
Thymol microparticles	Oral	5%	1–5 μm	Gastrointestinal infection targeting	Zhu et al. (2019)
Thymol polymer nanoparticles	Inhalation	1.5%	80–120 nm	Pulmonary antifungal therapy	Amato et al. (2016)

## 4 Thymol in Advanced Drug Delivery Platforms Microencapsulation, Nanoencapsulation and Lipid‐Based Delivery Systems

Over the past few years, microencapsulation as well as nanoencapsulation have been the focus of attention because of the highly successful improvements achieved in the stability, bioavailability, and release of thymol, one of the most unstable volatile monoterpenoid substances with diverse pharmacological applications. Spray drying is one of the most popular encapsulation routes for thymol because it is both economically and industrially viable. Rassu et al. (2014) successfully prepared spray‐dried microcapsules of methylcellulose and hydroxypropyl methylcellulose phthalate containing thymol as a tasteless oral formulation with retarded release in the gastrointestinal tract. The results showed that the methylcellulose matrix had a strong effect on the systemic absorption and bioavailability of thymol compared with the free one, whereas the hydroxypropyl methylcellulose phthalate system ensured localized delivery to the intestinal tract. In addition, nanoprecipitation techniques powered by biodegradable polymers such as chitosan have also shown enormous capabilities. (Zhao et al. 2023) prepared pH‐sensitive chitosan‐thymol nanoparticles with a loading capacity of 29.87% and an encapsulation efficiency of 41.92%. These nanoparticles were applied under controlled release conditions in vitro in an acidic solution and exhibited significantly enhanced antifungal activity against *Botrytis cinerea*, providing 78.73% protection in vivo compared with 61.13% with free thymol. The integration of pH‐responsiveness is one of the most important features of gastrointestinal delivery systems because thymol can be liberated by local pH changes, thereby facilitating therapeutic targeting and reducing off‐site effects. Coacervation is another potent technique for wrapping thymol. Charles et al. (2022) resorted to mucilage from yellow mustard and starch in emulsion electrospray to encapsulate thymol and carvacrol, obtaining encapsulation efficiencies ranging from 61.17% to 84.10%. These authors' nanocapsules presented multicore configurations, fewer air voids, and a release of even 120 h, in conformity with Fickian diffusion kinetics. A hypoallergenic application of the prolonged release profile can be achieved by the pharmaceutical and foodstuff sectors, particularly antimicrobial packaging and chronic therapy. In recent years, liposomal encapsulation has become an important method. According to Heckler et al. (2020), thymol enclosed in liposomes showed higher membrane permeability and stronger antimicrobial activity than free thymol. In general, these vesicular carriers provide biocompatibility as well as high encapsulation efficiencies that very often exceed the 75% mark and at the same time, they make it possible for thymol to be protected against oxidation as well as enzymatic degradation. Furthermore, nanoemulsion‐formulated thymol is more easily dispersed in water and is more bioavailable when administered orally. To obtain nanometric thymol emulsions, da Silva et al. (2023) used Tween 80 as the surfactant. The nanosystems had droplet sizes less than 150 nm and stable zeta potential values. As a result, increased antimicrobial activity against foodborne pathogens can be achieved. Nanoemulsions prepared using the high‐pressure homogenization technique or ultrasonication method permit accurate control of particle size and surface charge. These factors are critical for intestinal uptake and mucosal adhesion.

Sugiharto and Ayasan (2023) highlighted how micro‐ and nanoencapsulation of phytocompounds, such as thymol, could dramatically improve poultry nutrition. The bioavailability of nutrients in the encapsulated form along with the immune system response in broilers was greatly increased, whereas the need for synthetic growth promoters was notably lowered. This revelation proves the adaptable use of the encapsulated form of thymol in the pharmaceutical and animal health sectors. Gonçalves et al. (2017) prepared thymol‐loaded PLGA nanoparticles using a solvent evaporation technique. The particle size was ~170 nm, with a high loading capacity and continuous drug release after 72 h, as shown by in vitro gastrointestinal simulation. Anti‐inflammation was further enhanced in an in vivo study in rodents, and gastric irritation was reduced compared to free thymol. In addition, nanocapsules made of biopolymers are a notable approach. Zhang, Fu, et al. (2019) fabricated zein‐propylene glycol alginate nanoparticles for thymol encapsulation and achieved an encapsulation efficiency and physical stability of over 70%. The release behavior is dependent on the pH level; thus, the method of drug delivery is more practical in the intestine, where infections of the lower gastrointestinal tract can be addressed.

**FIGURE 6:**
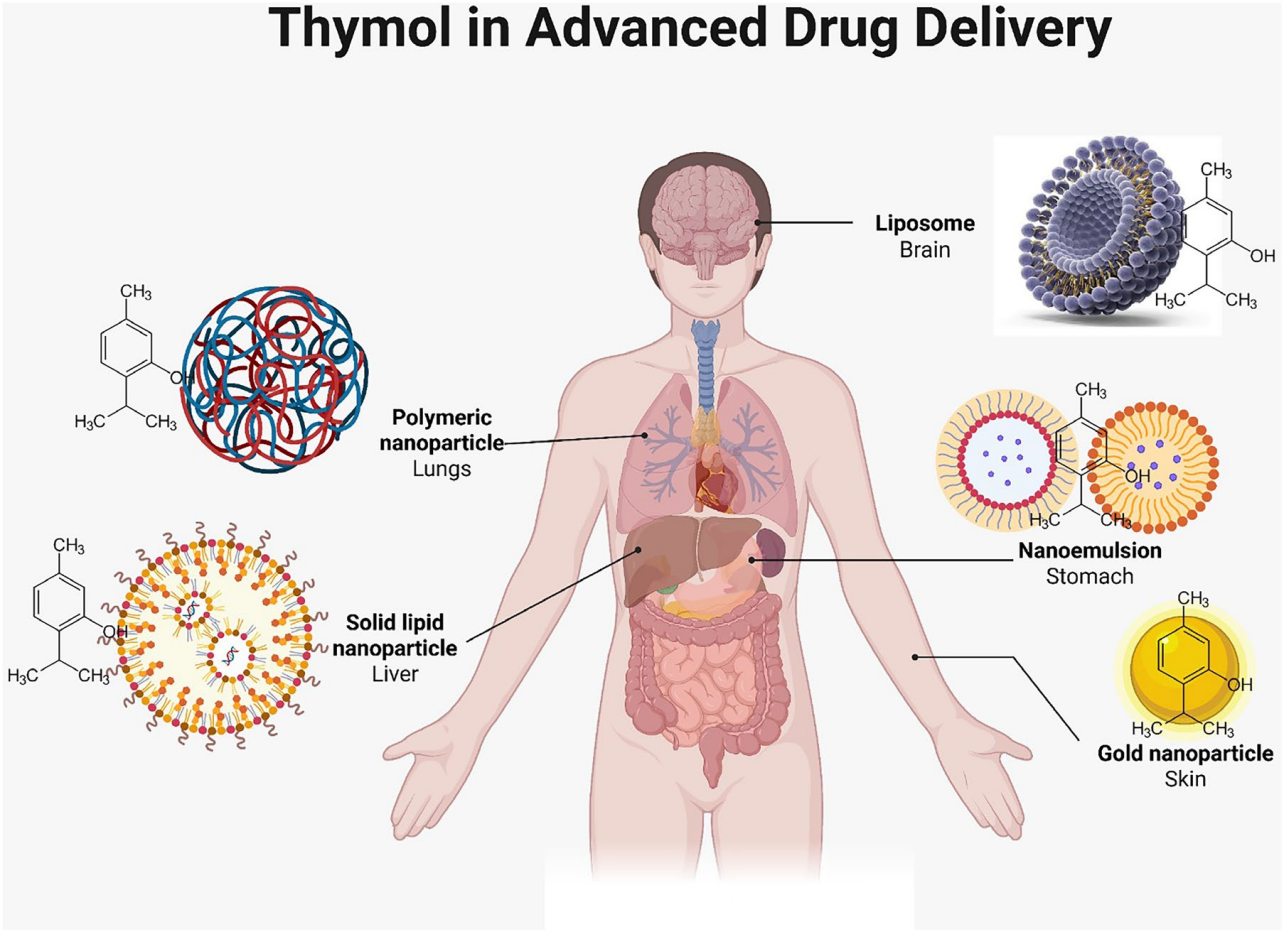
Site‐specific delivery of thymol via nanocarrier systems.

Nanostructured lipid carriers (NLCs) have become a prominent alternative for thymol delivery. Thymol‐loaded NLCs were prepared by (Folle et al. 2024) using glyceryl monostearate as the solid lipid and oleic acid as the oil; the particle size was lower than 200 nm, and the performance after 48 h of drug release was maintained. The newly created method of wound healing and topical antibacterial treatment has been proven to significantly outperform traditional creams. Finally, attention to the use of microfluidics versus traditional methods of thymol nanocarrier manufacturing is increasing. The above‐mentioned encapsulation methods collectively reflect substantial breakthroughs in circumventing the interference of thymol with the realization of its therapeutic function as planned. Material types, means of encapsulation, and release stimuli, especially pH, temperature, or enzymatic triggers, significantly affect the practical application of thymol, especially in the pharmaceutical, veterinary, and food sectors. Table 7 summarizes the newly emerged thymol encapsulation approaches, depicting the efficiency of loading, release, and therapeutic implications. A schematic of the organ‐specific delivery of thymol via nanocarriers is shown in Figure 6, which outlines the possibility of targeted therapeutic use.

**TABLE 7:** Thymol encapsulation strategies: Efficiency, particle size, and application profiles.

Encapsulation type	Loading efficiency (%)	Particle size (nm)	Release profile	Target/use	Study/author
Spray drying	85	190	Prolonged GI release	Oral, GI	Rassu et al. (2014)
Nanoprecipitation	42	220	pH‐triggered	Antifungal	Zhao et al. (2023)
Coacervation	84	150	Sustained (120 h)	Food/Pharma	Charles et al. (2022)
Liposomes	75	100	Stable + potent	Antimicrobial	Miranda‐Cadena et al. (2021)
PLGA nanoparticles	81	170	Slow release	Anti‐inflammatory	Gonçalves et al. (2017)
Biopolymer nanocapsules	70	180	Stable pH‐response	Intestinal release	Zhu et al. (2019)
Nanostructured lipid carriers (NLCs)	69	190	48 h sustained	Topical	Folle et al. (2024)
Solid lipid nanoparticles (SLNs)	66	175	48 h sustained	Topical	
Zein‐based nanoparticles	73	180	Intestinal‐specific	GI delivery	Zhu et al. (2019)

## 5 Multifunctional Applications of Thymol in Food Preservation

### 5.1 Antimicrobial and Antifungal Preservation

Thymol, a phenolic compound derived from thyme essential oil, has become a hot topic in food preservation owing to its strong antimicrobial and antifungal properties. Its effect is very powerful in various food matrices, such as meat, dairy, fruits, vegetables, and bakery products, indicating that it can act as a multifunctional agent. One of the main mechanisms by which thymol exerts its antimicrobial action is by damaging the outer membrane of microbial cells, which results in an increase in permeability, ion leakage, and cell lysis. Studies have found that thymol can reduce the microbial load of meat preservatives; thus, *Listeria monocytogenes* and *Escherichia coli* are the target pathogens. For example, (Mirsharifi et al. 2023) fabricated thymol‐chitosan films and applied them to chicken fillets, which led to alleviation of microbial counts and prolonged shelf‐life to 21 days compared to 6 days in the control group. Similarly, Zhang et al. (2022) revealed that thymol‐loaded zein nanofibers have great potential for the growth inhibition of *Salmonella typhimurium* in beef patties, thus maintaining color and texture during storage.

Thymol effectively inhibits the growth of yeasts and fungi in milk and dairy products, thereby enhancing microbial safety and extending shelf life. In this case, Escobar et al. (2020) tested the antifungal activity of thymol in yogurt and reported that a concentration of 0.02% was enough to completely inhibit the growth of *Candida kefyr* and *Aspergillus niger*. Thymol displayed a wider inhibition spectrum and relatively stable activity compared with potassium sorbate, the conventional preservative, under different pH values and storage conditions. Thymol, especially in the liposome carrier system, was observed to be more efficient in inhibiting milk emulsions than citrus oil, as reported by Sivaram et al. with an extension of storage at 4°C for fresh cheese. Therefore, thymol‐based edible coatings in fruits and vegetables have become one of the potential ways to counteract this delay in microbial decay (Sivaram et al. 2022). As a matter of fact, Oliveira et al. (2024) had a great example when he reported that the incorporation of thymol in alginate coatings applied to strawberries was able to inhibit the growth of *Botrytis cinerea* and *Penicillium expansum* significantly, which accounted for a 65% reduction in decay index and the maintaining of firmness for 10 days at refrigerated storage. Similarly, Pourhajibagher and Bahador (2021) discovered that fungal colonization was avoided by spraying thymol nanoemulsions on apples, and that the total phenolic content and antioxidant activity were retained much better than when using synthetic fungicides. Thus, the role of thymol and its ability to maintain nutritional quality were confirmed. Thymol vapor‐phase application on leafy greens not only prevented microbial proliferation but also extended visual quality, as reported by Murphy et al. (2022), who observed up to a 2 log CFU/g reduction in aerobic bacteria in spinach stored for 8 days. As for bakery products, thymol has attracted attention owing to its potential for the prevention of fungal spoilage and at the same time the maintenance of organoleptic properties. For instance, Safakas et al. (2025) studied the loading of thymo‐loaded biodegradable films into bread packaging and found that the texture and flavor were not affected at all, but at the same time *Aspergillus flavus* and *Penicillium chrysogenum* were suppressed to a significant extent. Abbaszadeh et al. (2014) also showed that the development of thymol in starch‐based coatings successfully decreased the production of mycotoxins in stored muffins, thereby offering a natural alternative to sodium benzoate. In addition, by combining thymol with other essential oil ingredients such as eugenol or carvacrol, thymol gave off stronger antifungal activity, providing the possibility for mixed preservative strategies. Numerous comparative studies of thymol and synthetic preservatives such as sodium nitrite, benzoates, and sorbates have been highlighted in the literature, with particular emphasis on the advantages of thymol's safety profile and environmentally friendly characteristics. One example is the systematic review by Sampaio et al. (2021), which supports that thymol retains an equivalent, if not superior, inhibition ability with very low cytotoxicity and resistance to development. In addition, certain encapsulation methods, such as nanoliposomes and solid lipid nanoparticles, have made it possible for thymol to be much more soluble in food matrices; the release of thymol is controlled, and its volatility is managed, as demonstrated by (Giotopoulou et al. 2024), particularly in preserved cheese and cold‐stored sausages of preserved cheese and cold‐stored sausages (Assaf and El Khatib 2021). The multifunctional role of thymol successfully exploits not only its powerful antimicrobial and antifungal activity but also its ability to extend product shelf life, retain sensory attributes, and provide a natural, consumer‐acceptable alternative to chemical preservatives, similar to this case of different food categories. As a result, the deployment of thymol in various foods highlights its wide‐ranging capabilities and possible further commercial uptake of this type of sustainable food preservation technology. Table 8 shows the practical application of thymol in different food products in combination with its antimicrobial efficacy.

**TABLE 8:** Applications of thymol in food preservation across diverse food products.

Food type	Thymol dose	Aim	Outcome	References
Fresh Beef	0.05% (w/w)	Reduce bacterial spoilage ( *E. coli* , *S. aureus* )	> 3 log CFU/g reduction	Zhang et al. (2022)
Yogurt	0.02% (free thymol)	Inhibit yeast & mold	Yeast/mold < 1 log CFU/mL	Singh et al. (2022)
Strawberries	0.05% thymol vapor	Prevent Botrytis cinerea	No fungal growth for 8 days	Zhang et al. (2023)
Tomato juice	0.15% thymol	Prevent fermentation	Total viable count < 2 log	Lee et al. (2020)
Blueberries	0.1% thymol	Prevent Rhizopus & Penicillium	No fungal growth for 7 days	Ye et al. (2023)
Mayonnaise	0.05% thymol	Inhibit yeasts	90% inhibition at 10°C	Zhang et al. (2023)
Egg wash	0.15% thymol	Decontaminate shell eggs	99.9% Salmonella reduction	Jin et al. (2013)
Pasta	0.05% thymol	Control spoilage	No mold, good texture	Bonomi et al. (2012)
Lettuce	0.1% thymol wash	Improve microbial safety	2.7 log CFU/g reduction	Hashemi et al. (2023)
Cucumber	0.15% thymol + packaging	Inhibit *E. coli*	Counts < 2 log	Singh and Maurya (2023)
Cake	0.1% thymol	Prevent spoilage	Mold‐free 5 days	Sharma et al. (2024)
Fresh corn	0.2% thymol	Enhance storage	4 log microbial reduction	Xue et al. (2022)
Almond milk	0.15% thymol	Maintain quality	TBARS reduced 35%	Chen et al. (2023)

### 5.2 Active and Intelligent Packaging

Thymol has become the most important one among the bioactive compound in active and intelligent food packaging because of its radical antimicrobial, antioxidative, and volatile nature. These features allow consumers not only to keep fruits and vegetables for a longer period of time but also to extend the shelf life of these products by providing the so‐called shelf life while reducing the use of synthetic additives. Influential packaging systems that insert thymol deeply into polymer matrices, such as chitosan, gelatin, and polyvinyl alcohol (PVA), have been extensively shown to suppress microbial activity. For instance, Mohajer et al. (2023) proposed thymol‐charged chitosan films, which notably decreased the number of *Escherichia coli* and *Listeria monocytogenes* in fresh‐cut lettuce and attained a microbial reduction of over 3 log CFU/g along with a shelf‐life increment of 10 days under refrigeration. Zhang, Zhang, Bao, et al. (2024) studied the process of loading thymol into sodium alginate films through emulsification, which yielded 82% inhibition of *Penicillium expansum* in strawberries and retention of firmness and antioxidant capacity over 12 days of storage. Karimi‐Khorrami et al. (2022) produced starch‐based films loaded with thymol nanoemulsions, in which the release was controlled, and the bacterial counts in cheese were reduced by 4.2 log units. The nanodispersion method not only allowed for a better distribution within the polymer matrix but also improved the barrier properties owing to the smaller droplet size and increased surface activity. In contrast, intelligent packaging with thymol as a sensor or indicator of freshness is a new trend in the development of the field. Roy et al. (2024) reported pH‐responsive thymol‐impregnated gelatin films that merged red cabbage anthocyanin indicators. These colorimetric films allow the consumer to visually see the meat freshness level by the existing color changes rapidly as the pH increases. This development demonstrated not only the benefit of thymol as an antimicrobial agent being merged with color‐changing intelligent systems but also the ability to perform multifunctional smart packaging as multifunctional.

**FIGURE 7:**
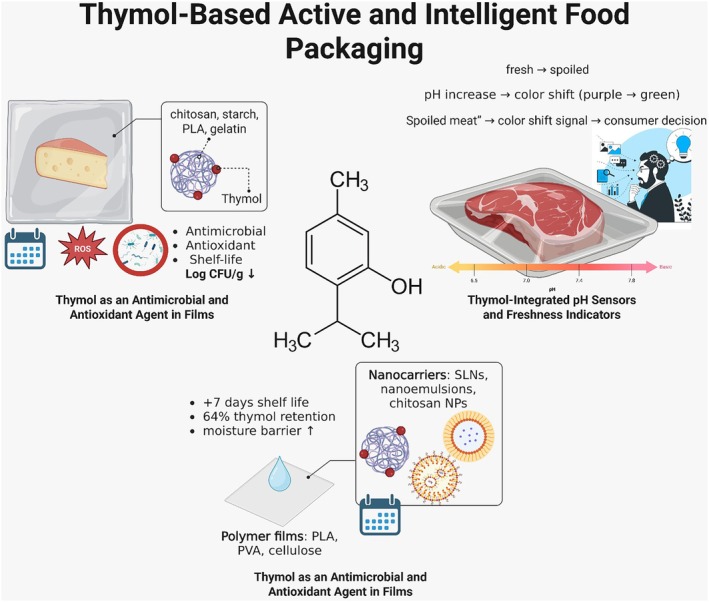
Thymol‐based active and intelligent food packaging systems.

The application of nanostructured carriers has brought major improvements to the stability and kinetics of thymol release in packaging films. More specifically, the study of Ab Rashid et al. (2023) has already been discussed in addition to embedded solid lipid nanoparticles (SLNs) loaded with thymol into PVA films, which led to the release over 15 days increased by 28%, accompanied by a significant antimicrobial activity on *S. aureus* and *S. typhimurium* at the same concentrations. Moreover, to prepare cellulose‐based nanofibers containing thymol, the study developed a packaging material which extended the shelf life of chicken breast up to 7 days without odor perception (Zainal et al. 2023). Another research demonstrated that baking products coated with the same PLA‐films along with thymol could decrease *Aspergillus niger* growth by 98% without harming sensory properties of the samples. Moreover, their films also displayed a Fickian diffusion release pattern leading to long‐term microbial inhibition (Mohamad et al. 2020). Figure 7 illustrates the role of thymol in active and intelligent packaging systems including antimicrobial films and pH‐responsive indicators for spoilage detection.

Moreover, the presence of thymol and zinc oxide nanoparticles in the structure of biodegradable intelligent films results in microbial inhibition, in addition to the good barrier properties of the films against moisture and oxygen (Kazemi‐Pasarvi et al. 2020; Shankar et al. 2018). This dual characteristic supports the preservation and integrity of packaging over long periods. Similarly, Ramos et al. (2020) created PLA films containing thymol, curcumin‐loaded nanocarriers, and boasted antioxidant, antimicrobial, and smart color‐shift properties for meat applications. These results strongly confirm the multifunctional role of thymol as a handle for future food packaging. The use of thymol in both active and intelligent packaging allows for microbial protection and spoilage sensing at the same time; thus, food waste is reduced to a great extent, while both the safety and quality of food products are maintained. Further progress in polymer compatibility, nanotechnology, and encapsulation strategies will facilitate the sustainable migration of thymol from laboratory work to commercial food packaging platforms.

## 6 Toxicological and Safety Considerations

Thymol is a monoterpenoid phenol found in large quantities in the essential oils of thyme and oregano. It has attracted attention primarily because of its antimicrobial and antioxidant properties. Nevertheless, the toxicological and safety considerations of thymol are required for its application in food or pharmaceutical products. Studies on acute toxicity have shown that thymol, when taken orally at moderate doses, is generally nontoxic. The research result on the LD50 of thymol in rats is approximately 980 mg/kg body weight, thus categorizing it as a substance with low toxicity (Gad et al. 2013). Similarly, Gad et al. (2013) indicated that the cytotoxicity of thymol in the nanoemulsion form was lowered, while the bioavailability was improved, and no symptoms of acute toxicity were observed in animal models. The phenol study by Cohen et al. (2021) included compounds such as thymol and carvacrol, which are in agreement with the “Generally Recognized as Safe” (GRAS) status given by the Flavor and Extract Manufacturers Association (FEMA). The tested compounds did not show potential mutagenic, carcinogenic, or teratogenic effects in the applied toxicologically safe screenings. This impression is reinforced by the European Food Safety Authority (EFSA) that sanctioned thymol as a flavoring agent in food products without concentration exceeding 0.5 mg/kg of the final product (EFSA Panel on Food Additives and Nutrient Sources added to Food (ANS) 2012). These pharmacokinetic studies show that thymol is quickly absorbed and metabolized, mostly in the liver, and excreted mainly as glucuronide and sulfate conjugates via urine. The metabolic clearance of the molecule is efficient, and there is no tissue accumulation; consequently, long‐term toxicity is low, as reported by (Randhawa and Alghamdi 2011). Besides that, the experimental results of the study by Rodriguez‐Garcia et al. (2016) did not show any significant changes in the blood or liver parameters of the rats after oral thymol supplementation at 100 mg/kg/day for 28 days. Nevertheless, the source of this problem is at concentrations higher than the recommended concentrations. As the point they made, Epps et al. (2015) show that thymol and its glucoside derivative are the origin of these problems such as gastrointestinal disturbances and mucosal irritation if the dosage is higher than 200 mg/kg, especially in poultry feed. Additionally, a study by Samah R et al. (2023) on the immunomodulatory potential of thymol in zebrafish suggested slight changes in immune gene expression occurring at concentrations above 150 mg/L in aquatic environments; therefore, dose optimization is fundamental.

In terms of food applications, thymol‐loaded edible films applied to fruits and dairy products have been demonstrated by Sivaram et al. (2022) to be safe, as they do not release compounds in the food matrix in quantities that exceed the acceptable daily intake (ADI) limits. In addition, cellular tests similar to those accomplished by Llana‐Ruiz‐Cabello et al. (2014) with human fibroblasts and Caco‐2 cell lines have pointed out the biocompatibility of thymol, with no cytotoxic effects observed at doses lower than 50 μM. Moreover, in vivo experiments on rodents by Aboushouk et al. (2021) revealed that thymol did not cause histopathological alterations in the liver, kidney, and spleen tissues even after 90 days of oral administration. Overall, the compilation of studies largely supports the claim that thymol can be allowed in food and drug preparations, provided it is within the prescribed limits. The product has low acute and chronic toxicity, efficient metabolic elimination, and GRAS status according to both the FDA and EFSA under certain conditions. However, there should be a watchful eye on new delivery systems such as nanocarriers or inclusion in complex food matrices to ensure that they are safe for all demographics and exposure profiles.

## 7 Conclusion

Thymol has emerged as a multifunctional natural compound with considerable potential in pharmaceutical and food applications, owing to its antimicrobial, anticancer, antioxidant, and anti‐inflammatory properties. Its mechanisms of action, including membrane disruption, oxidative stress modulation, and regulation of cellular signaling pathways, highlight its versatility as both a therapeutic agent and a natural preservative. Despite these advantages, several limitations hinder its broader application. In particular, thymol's low aqueous solubility, high volatility, and physicochemical instability significantly affect its bioavailability and therapeutic performance. Although advanced delivery systems such as nanoemulsions, liposomes, and polymeric carriers have demonstrated promising improvements in stability and controlled release, challenges related to scalability, cost, and regulatory approval remain unresolved. Future research should focus on developing cost‐effective and scalable delivery platforms, improving formulation stability, and conducting long‐term in vivo and clinical studies. Addressing these challenges will be essential to fully exploit thymol's potential and facilitate its translation into sustainable and effective applications in both medicine and food preservation systems.

## Author Contributions

**Farhang Hameed Awlqadr:** conceptualization, methodology, software, data curation, resources, writing – review and editing, writing – original draft, supervision, validation. **Mohammed N. Saeed** conceptualization, methodology, software, data curation, resources, writing – review and editing, writing – original draft, supervision, validation. **Ammar B. Altemimi:** conceptualization, methodology, software, data curation, resources, writing – review and editing, writing – original draft, supervision, validation. **Syamand Ahmed Qadir:** writing – original draft, writing – review and editing, conceptualization, methodology, software, resources, validation, data curation, supervision. **Aryan Mahmood Faraj:** writing – original draft, writing – review and editing, conceptualization, methodology, software, resources, validation, data curation, supervision. **Othman Abdulrahman Mohammed:** writing – original draft, writing – review and editing, conceptualization, methodology, software, resources, validation, data curation, supervision. **Tablo H. Salih:** writing – original draft, writing – review and editing, conceptualization, methodology, software, resources, validation, data curation, supervision. **Rawaa H. Tlay:** writing – original draft, writing – review and editing, conceptualization, methodology, software, resources, validation, data curation, supervision. **Tarek Gamal Abedelmaksoud:** writing – original draft, writing – review and editing, conceptualization, methodology, software, resources, validation, data curation, supervision.

## Funding

The authors have nothing to report.

## Ethics Statement

The authors have nothing to report.

## Conflicts of Interest

The authors declare no conflicts of interest.

## Data Availability

The data that support the findings of this study are available from the corresponding author upon reasonable request.
